# High Rates of Missed HIV Testing Among Oral PrEP Users in the United States From 2018–2021: A National Assessment on Compliance With HIV Testing Recommendations of the CDC PrEP Guidelines

**DOI:** 10.1093/ofid/ofae254

**Published:** 2024-05-23

**Authors:** Jason Baron, Tamar Tchelidze, Benjamin LaBrot, Joseph Yao, Kenneth H Mayer, Daniel Kuritzkes, Nicole Robinson, Rupa R Patel

**Affiliations:** Roche Diagnostics, Indianapolis, Indiana, USA; Roche Diagnostics, Indianapolis, Indiana, USA; Roche Molecular Systems, Pleasanton, California, USA; Mayo Clinic, Rochester, Minnesota, USA; Fenway Health and Harvard Medical School, Boston, Massachusetts; Brigham and Women's Hospital and Harvard Medical School, Boston, Massachusetts, USA; Roche Diagnostics, Indianapolis, Indiana, USA; Washington University School of Medicine, St Louis, Missouri, USA

**Keywords:** HIV testing, laboratory utilization, PrEP, quality of care, pre-exposure prophylaxis

## Abstract

**Background:**

The US Centers for Disease Control and Prevention recommends HIV testing every 3 months in oral PrEP users. We performed a national assessment of HIV testing compliance among oral PrEP users.

**Methods:**

We analyzed 408 910 PrEP prescriptions issued to 39 809 PrEP users using a national insurance claims database that contained commercial and Medicaid claims. We identified PrEP use based on pharmacy claims and outpatient diagnostic coding. We evaluated the percentage of PrEP prescription refills without HIV testing (identified by CPT codes) within the prior 3, 6, and 12 months using time to event methods. We performed subgroup and multivariate analyses by age, gender, race, insurance type, and geography.

**Results:**

Of 39 809 persons, 36 197 were commercially insured, 3612 were Medicaid-insured, and 96% identified as male; the median age (interquartile range) was 34 (29–44) years, and the Medicaid-insured PrEP users were 24% Black/African American, 44% White, and 9% Hispanic/Latinx. Within the prior 3, 6, and 12 months, respectively, the percentage of PrEP prescription fills in individuals without HIV Ag/Ab testing was 34.3% (95% CI, 34.2%–34.5%), 23.8% (95% CI, 23.7%–23.9%), and 16.6% (95% CI, 16.4%–16.7%), and the percentage without any type of HIV test was 25.8% (95% CI, 25.6%–25.9%), 14.6% (95% CI, 14.5%–14.7%), and 7.8% (95% CI, 7.7%–7.9%).

**Conclusions:**

Approximately 1 in 3 oral PrEP prescriptions were filled in persons who had not received an HIV Ag/Ab test within the prior 3 months, with evidence of health disparities. These findings inform clinical PrEP monitoring efforts and compliance with national HIV testing guidance to monitor PrEP users.

Despite great advances in HIV prevention and treatment, national HIV incidence remains notable, with >32 000 infections per year in the United States [1]. The US Centers for Disease Control and Prevention (CDC) has set a target of reducing transmission by 90% by 2030 [2]. Both pharmacological and nonpharmacological strategies remain critically important in achieving this goal and associated gains in public health. One crucial tool for reducing HIV transmission is oral HIV pre-exposure prophylaxis (PrEP) [3, 4]. PrEP involves use of antiretroviral medications before potential HIV exposure [4]. Two combined formulations of emtricitabine/tenofovir (tenofovir disoproxil and emtricitabine [Truvada] and tenofovir alafenamide and emtricitabine [Discovy]) are currently approved for oral PrEP in the United States. In addition to these oral formulations, long-acting cabotegravir (CAB-LA) can be used for injectable PrEP.

PrEP users need to be tested for HIV infection at regular intervals [5]. Since 2021, the CDC has recommended combined HIV antigen/antibody (Ag/Ab) testing and HIV nucleic acid amplification testing (NAAT) for quarterly monitoring in PrEP users taking daily oral medications [6]. Through 2020 (for which most of the data for this paper were extracted), the requirement for NAAT testing was not included, but every-3-month HIV testing was recommended with a preference for Ag/Ab tests [7]. HIV testing during PrEP use is critical to ensure that persons taking PrEP continue to be HIV-uninfected after initiation and to avoid selecting for resistant strains if the PrEP user becomes HIV-infected while using PrEP. In the case of HIV infection during PrEP use, medications need to be changed to a regimen that provides treatment. Continuing PrEP after acquiring HIV can lead to delayed treatment, resistance-associated mutations, clinical morbidity and mortality, and HIV transmission [5].

The current literature assessing the fidelity to national guidelines for HIV testing during PrEP use is limited. Data are needed regarding HIV testing patterns to identify areas of noncompliance and develop targeted public health and quality improvement initiatives. McCormick et al. found that only 6.4% of PrEP physician prescribers were providing care that met their definition of high quality with regard to laboratory testing. Similarly, Huang et al. found suboptimal testing among US PrEP users from 2011 to 2015 [8]. We sought to examine more recent HIV testing patterns among those prescribed PrEP, stratified by HIV test type and PrEP user characteristics, to inform clinical and public health programs.

## METHODS

We conducted an analysis of 408 910 PrEP prescription fills issued to 39 809 PrEP users between 2018 and 2021 using MarketScan, a national insurance claims database for private and public payors.

The primary outcome was the number of calendar months between each PrEP medication fill and the PrEP user's most recent prior HIV Ag/Ab test. Secondary outcomes included HIV testing patterns (with antibody-only and NAAT testing) and sociodemographic and geographic differences.

### Data Set

We retrospectively extracted study data from the MarketScan national insurance claims databases [9] for commercially insured (n = 36 197 unique persons) and Medicaid-insured (n = 3612) persons. MarketScan has been used in prior academic and clinical research including the study of PrEP [10–15]. The data set included claims for outpatient services and prescription medications (pharmacy). The database for commercially insured persons includes individuals with employer-sponsored health insurance (including employees, dependents, and retirees) from participating payors, with reportedly >350 payors represented from across all 50 states. The Medicaid database includes individuals with Medicaid insurance from “multiple states” (with specific states not disclosed). (Database descriptions were based on the 2021 Marketscan user guide and personal experience with the data.) In addition, for nearly all individuals, the database included birth year and reported gender. It also included race for Medicaid individuals and geography (ie, metropolitan statistical area [MSA] and US region) for commercially insured individuals. Among other attributes, outpatient services claims included diagnosis (usually International Classification of Diseases [ICD]–10) [16] and procedure (Healthcare Common Procedure Coding System [HCPCS]/Current Procedural Terminology [CPT]) [17] codes; pharmacy claims included National Drug Codes (NDC) [18] indicating the specific medication dispensed. We included only individuals with PrEP prescriptions between 2018 and 2021. To improve the sensitivity of our exclusion criteria (below) and to capture HIV testing preceding PrEP prescriptions, we included data points related to exclusion criteria and HIV testing as far back as 2016.

### Inclusion Criteria and Definition of PrEP

Inclusion criteria were PrEP prescriptions filled from 2018 to 2021. PrEP use was defined as 2 or more prescription fills in different calendar months for antiretrovirals (ARVs) approved for oral PrEP in HIV-negative persons who had no additional ARV prescriptions. In particular, paralleling prior studies using Marketscan to study PrEP, we adapted 4 criteria to define PrEP use. Individuals in the database were considered PrEP users if:

- they filled at least 2 prescriptions (in distinct calendar months) for emtricitabine/tenofovir (ie, FTC/TDF and FTC/TAF), identified using the NDC codes described in Supplementary Appendix A, between 2018 and 2021. The 2 prescription criteria were adapted from prior work and were in part intended to exclude individuals on postexposure prophylaxis (PEP) who would only get 1 prescription for antiretroviral medication (if no subsequent PEP, referral for PrEP or HIV acquisition).
- they did not have a diagnosis of HIV at any point between 2016 and 2021 in the outpatient services table based on the ICD-10 codes in Supplementary Appendix A.
- they did not have a diagnosis of hepatitis B virus (HBV) at any point between 2016 and 2021 in the outpatient services table based on the ICD-10 codes in Supplementary Appendix A. HBV could represent an alternative explanation for emtricitabine/tenofovir use, and therefore only individuals without HBV were included.
- they must not have been on other antiretrovirals between 2016 and 2021 as indicated in the outpatient pharmacy claims tables based on NDC codes defined in Supplementary Appendix A; this criterion allowed exclusion of those with active HIV infection.

**Figure 1.**
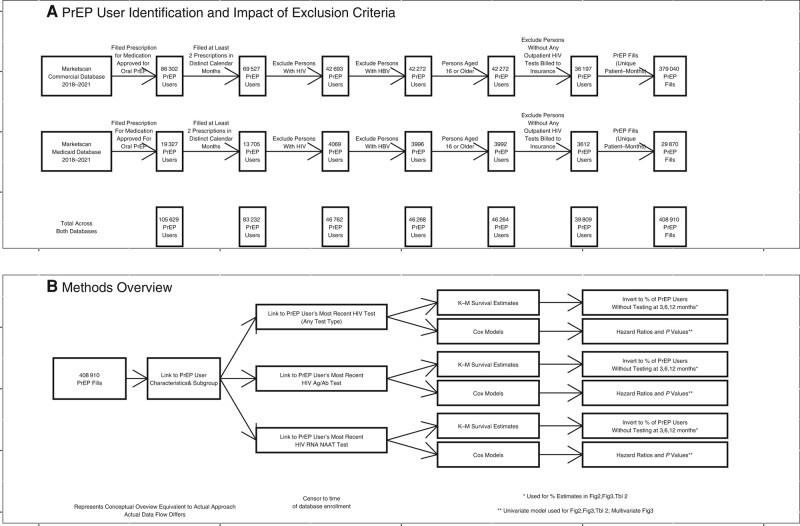
Inclusion criteria, sample sizes, and data flow. *A*, Inclusion criteria and their impact on the sample of PrEP prescriptions included in the analysis. *B*, Overview of the methods and data flow. Abbreviations: Ag/Ab, antigen/antibody; HBV, hepatitis B virus; PrEP, pre-exposure prophylaxis.

We additionally excluded PrEP users who did not have at least 1 HIV test of any type in the database (2016–2021). We developed this exclusion criterion to exclude PrEP users who were likely receiving PrEP care in settings that did not bill insurance. Figure 1 provides an overview of our inclusion/exclusion criteria, the number of persons included or excluded by each criterion, and a general overview of our data flow and methods.

### Time to HIV Testing and Censoring

We calculated the number of calendar months between each PrEP prescription and the PrEP user's most recent prior HIV test. For these purposes, a test in the calendar month in which the PrEP prescription was filled would be considered 0 months prior; the prior calendar month would be considered 1 month prior, and so forth. We timestamped PrEP prescription fills and HIV tests to the calendar month in which they occurred instead of actual dates for consistency with coverage information. Resolving time to the calendar month may not always reflect exact time. However, our time accounting resolves any ambiguity-related use of calendar months and not actual days in favor of assuming that testing was performed within various time windows and is thus “conservative.” Individuals were censored to the time of first continuous enrollment in the database if they did not have a known HIV test before the PrEP prescription fill (Supplementary Appendix A).

### Definitions of HIV Test Types

We defined HIV testing using CPT codes, as shown in Supplementary Appendix A. For certain analyses, we considered subtypes of HIV testing (ie, combined HIV Ag/Ab tests, HIV RNA NAAT tests, and HIV Ab-only tests). Tests not billed to insurance (including some rapid or at-home tests) would not be captured by MarketScan data and thus are not included in this analysis. In some of the analyses, we looked at only certain types of HIV tests (ie, combined HIV Ag/Ab tests, HIV RNA NAAT tests); for these analyses, we only considered those tests billed with the corresponding CPT codes (Supplementary Appendix A) and disregarded any other HIV testing.

### Definitions of PrEP User Characteristics

We captured the PrEP user's age, gender, race, region, and MSA as defined in Supplementary Appendix A. Geography was not available for Medicaid persons, and race was not available for commercially insured persons. We grouped MSAs into combined metropolitan statistical areas (CBSAs) using a crosswalk available from census.gov and analyzed data by CBSA to explore differences between metropolitan areas.

For each PrEP prescription fill, we calculated the number of continuous months the PrEP users had been on PrEP as the number of calendar months since the PrEP user's earliest PrEP prescription without a break in prescription filling of at least 2 calendar months.

### Patient Consent

Because only fully de-identified data were used in this study, this study did not constitute human subjects research, and institutional review board approval was deemed to not be needed.

### Statistical Analysis and Plotting

The time (number of calendar months) between each PrEP prescription fill and the PrEP user's most recent prior HIV test was modeled using time to event methods (equivalent to “survival” modeling) [19]. Time since most recent test was censored to the time of enrollment in the database. Kaplan-Meier methods were used to estimate the actual percentage and CI for the proportion of PrEP users getting tested within a specified time frame of the PrEP prescription refill. Cox proportional hazard models were used to estimate *P* values, hazard ratios, and CIs for hazard ratios in subgroup and multivariate analyses. *P* values comparing hazard ratios used a null hypothesis that the hazard ratio equaled 1; *P* values <.05% and 95% CIs around hazard ratios that did not cross 1 were considered statistically significant.

Statistical analysis was performed in R studio [20]. Survival modeling used the R package “survival.” Plots were performed in R using ggplots. Geographic mapping was performed using latitude and longitude coordinates, as described in Supplementary Appendix A.

## RESULTS

The final data set included 408 910 PrEP prescription fills among 39 809 unique PrEP users. As shown in Table 1, of the 39 809 PrEP users, 36 197 had private and 3612 had public (Medicaid) insurance, 96% identified as male, and the median age (interquartile range [IQR]) was 34 (29–44) years. Ninety-seven point six percent of commercially insured PrEP users identified as male. Commercially insured PrEP users had a median age (IQR) of 35 (29–44) years, with 2.7% living in rural areas, and were regionally distributed throughout the United States. Medicaid-insured users had a median age (IQR) of 32 (26–40) years; 82.7% identified as male, 23.6% as Black/African American, 8.9% Hispanic/Latinx, 44.2% White, and 16.4% other races.

**Table 1.** Characteristics of Persons Using PrEP

Description	Commercial Insurance	Medicaid	Combined
Unique PrEP users	36 197	3612	39 809
PrEP prescription fills	379 040	29 870	408 910
Prescription fills per prep user Median (IQR)	8 (4–14)	5 (3–11)	7 (4–14)
Age Median (IQR)	35 (29–44)	32 (26–40)	34 (29–44)
% male	97.6	82.7	96.2
% rural	2.7	NA	NA
Race (Medicaid-only), %			
Black/African American	NA	23.6	NA
Hispanic/Latinx	NA	8.9	NA
Other races	NA	16.4	NA
White	NA	44.2	NA
Region (commercial insurance only), %			
North Central	14	NA	NA
Northeast	26.4	NA	NA
South	36.6	NA	NA
West	21.6	NA	NA

Note that percentages within a category do not necessarily sum to 100% due to cases where the attribute was unknown.

Abbreviations: IQR, interquartile range; PrEP, pre-exposure prophylaxis.

**Figure 2.**
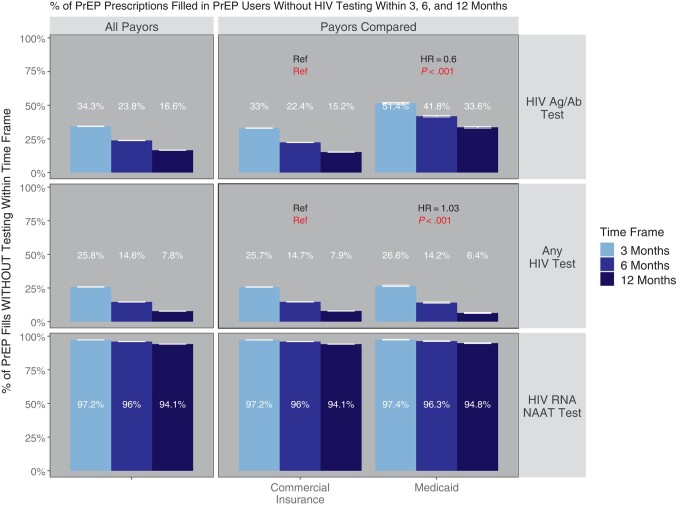
Percentage of PrEP medication fills without HIV testing within 3, 6, and 12 months. Shown is the proportion of PrEP prescriptions with HIV testing of the specified type within 3, 6, or 12 months before the PrEP prescription fill, subgrouped by insurance type. *P* values and hazard ratios compare each subgroup of PrEP users with the reference category (“ref”) in each cell; HRs >1 indicated a greater propensity to have testing within a given time frame in comparison with the reference (and vice versa for HR <1). *P* values compare the hazard ratios against a null hypothesis of HR = 1. Error bars reflect 95% CIs. Abbreviations: Ag/Ab, antigen/antibody; HR, hazard ratio; NAAT, nucleic acid amplification testing; PrEP, pre-exposure prophylaxis.

We found that 34.3% (95% CI, 34.2%–34.5%), 23.8% (95% CI, 23.7%–23.9%), and 16.6% (95% CI, 16.4%–16.7%) of PrEP prescriptions were filled in persons who had not received HIV Ag/Ab testing within the prior 3, 6, and 12 months, respectively (CIs account for sampling) (Figure 2). In a subgroup analysis by payor (Figure 2), 33% (95% CI, 32.8%–33.1%), 22.4% (95% CI, 22.2%–22.5%), and 15.2% (95% CI, 15.1%–15.3%) of PrEP fills in commercially insured PrEP users and 51.4% (95% CI, 50.9%–52%), 41.8% (95% CI, 41.2%–42.4%), and 33.6% (95% CI, 33%–34.2%) of fills in Medicaid-insured PrEP users were not associated with HIV Ag/Ab testing within 3, 6, and 12 months, respectively (HR, 0.60; *P* < .001). Twenty-five point eight percent (95% CI, 25.6%–25.9%), 14.6% (95% CI, 14.5%–14.7%), and 7.8% (95% CI, 7.7%–7.9%) of PrEP prescriptions were filled in individuals who had not received any type of HIV test (including Ag/Ab, Ab-only, and NAAT) in the prior 3, 6, and 12 months, respectively. Ninety-seven point two percent (95% CI, 97.1%–97.2%), 96% (95% CI, 95.9%–96.1%), and 94.1% (95% CI, 94%–94.2%) of PrEP fills were not associated with HIV RNA NAAT testing within the prior 3, 6, and 12 months, respectively. Of note, while Medicaid PrEP users had less HIV Ag/Ab testing (HR, 0.6; 95% CI, 0.59–0.61; *P* < .001), there were only trivial differences (HR, 1.03; 95% CI, 1.01–1.04), albeit statistically significant (*P* < .001), between types in terms of rates of any HIV test, suggesting that overall testing rates are similar but that Medicaid-insured PrEP users were less likely to receive the guideline-recommended HIV Ag/Ab tests.

We also performed a confirmatory analysis paralleling that shown in Figure 2 but with a single PrEP refill sampled per PrEP user (Supplementary Figure 1). This confirmatory analysis found that the primary results were not unduly biased because PrEP prescription fills were not entirely independent to the extent that they were linked by the PrEP user and the same HIV test may serve as the most recent for multiple PrEP prescription fills. The results of this confirmatory analysis were highly consistent with and support our primary findings.

**Figure 3.**
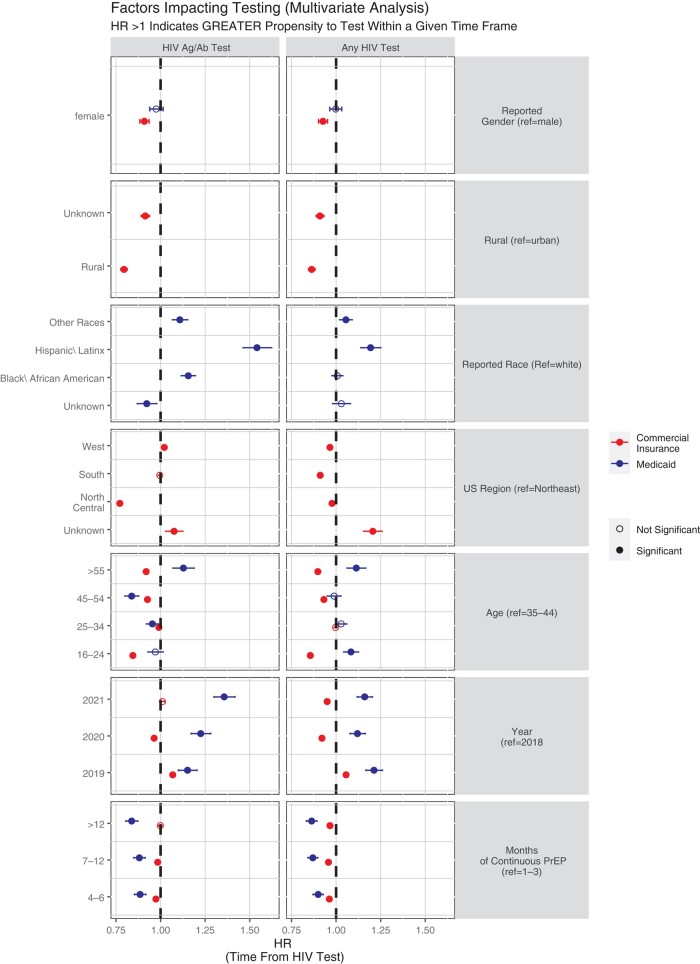
Factors impacting HIV testing in multivariate analysis. Shown are results from a multivariate analysis examining factors that affect the propensity of a PrEP user to have HIV testing (any type of HIV test, right column, or HIV Ag/Ab test, left column). HRs >1 indicated a greater propensity to have received HIV testing within a given time frame in comparison with the reference, specified as “ref.” Error bars (reflecting 95% CIs) not crossing the dashed vertical lines at HR = 1 signal statistical significance (statistical significance is indicated by filled points). Abbreviations: HR, hazard ratio; PrEP, pre-exposure prophylaxis.

Subgroup analyses by PrEP user characteristics revealed that in the commercially insured population, rural users were less likely to be tested with HIV Ag/Ab tests (HR, 0.89; *P* < .001), as were PrEP users in the Northcentral and South United States (HR, 0.96; *P* < .001 for both Northcentral and South; ref = Northeast) (Table 2). In the Medicaid-insured population, male PrEP users had more HIV Ag/Ab testing than females (HR, 0.96; *P* = .034; ref = male), and Hispanic/Latinx PrEP users had more HIV Ag/Ab testing (HR, 1.19; *P* < .001) compared with White PrEP users. Multivariate analyses (Figure 3) revealed that Hispanic/Latinx and Black/African American PrEP users were more likely to have HIV Ag/Ab testing when adjusting for sex, age group, calendar year, and months on PrEP. Rural PrEP users and PrEP users in the Northcentral United States as well as PrEP users who had been on PrEP for >3 months generally had less HIV Ag/Ab testing and testing of any type; the findings were not statistically significant in the case of commercially insured PrEP users on PrEP for >12 months, and the effect of months on PrEP was only modest in the commercially insured population with respect to HIV Ag/Ab testing.

**Table 2.** Proportion of PrEP Prescriptions Filled Without HIV Testing at 3, 6, and 12 Months by PrEP User Subgroup

			HIV Ag/Ab Test				Any HIV Test			
			% With No Testing			HR	% With No Testing			HR
Data Set	Category	Variable	3 mo	6 mo	12 mo		3 mo	6 mo	12 mo	
Commercial insurance	Calendar year	2018	30 (29.7–30.3)	21.1 (20.9–21.4)	15.1 (14.8–15.3)	Ref Ref	22.5 (22.2–22.7)	13.1 (12.9–13.3)	7.4 (7.2–7.6)	Ref Ref
		2019	27.9 (27.7–28.2)	18.9 (18.7–19.1)	13 (12.8–13.2)	HR = 1.06 *P* < .001	21 (20.8–21.2)	11.8 (11.6–12)	6.4 (6.3–6.6)	HR = 1.04 *P* < .001
		2020	33.1 (32.8–33.4)	21.7 (21.5–22)	13.9 (13.7–14.2)	HR = 0.98 *P* < .001	26.7 (26.4–27)	14.9 (14.7–15.1)	7.7 (7.6–7.9)	HR = 0.93 *P* < .001
		2021	28.9 (28.6–29.2)	19.8 (19.5–20.1)	13.5 (13.3–13.8)	HR = 1.08 *P* < .001	23.7 (23.4–24)	14.3 (14.1–14.6)	8.3 (8.2–8.5)	HR = 0.99 *P* = .002
	Setting	Urban	29.7 (29.6–29.8)	20.1 (20–20.2)	13.6 (13.5–13.8)	Ref Ref	23.2 (23.1–23.3)	13.3 (13.2–13.4)	7.3 (7.2–7.4)	Ref Ref
		Rural	37 (36.1–37.9)	27.6 (26.8–28.5)	20.5 (19.7–21.4)	HR = 0.82 *P* < .001	27.9 (27–28.7)	17.3 (16.6–18.1)	10.2 (9.6–10.9)	HR = 0.89 *P* < .001
	Region	Northeast	29.8 (29.5–30.1)	19.5 (19.2–19.7)	12.6 (12.4–12.8)	Ref Ref	22.6 (22.3–22.8)	12 (11.8–12.2)	6 (5.8–6.1)	Ref Ref
		North Central	37.4 (37–37.8)	27.9 (27.6–28.3)	21.4 (21.1–21.8)	HR = 0.77 *P* < .001	23.8 (23.5–24.2)	13.3 (13.1–13.6)	7 (6.8–7.2)	HR = 0.96 *P* < .001
		South	28.4 (28.2–28.6)	19.1 (18.9–19.3)	12.7 (12.5–12.9)	HR = 1.05 *P* < .001	24 (23.8–24.2)	14.5 (14.3–14.6)	8.4 (8.2–8.5)	HR = 0.96 *P* < .001
		West	27.8 (27.5–28.1)	18.6 (18.3–18.9)	12.4 (12.2–12.6)	HR = 1.06 *P* < .001	22.8 (22.5–23.1)	13.4 (13.2–13.7)	7.6 (7.4–7.8)	HR = 1 *P* = .568
	Age	35–44 y	28.7 (28.4–28.9)	19 (18.8–19.3)	12.9 (12.7–13.1)	Ref Ref	22.5 (22.3–22.8)	12.5 (12.3–12.7)	6.6 (6.4–6.7)	Ref Ref
		16–24 y	32.9 (32.3–33.5)	24.2 (23.6–24.8)	17.3 (16.8–17.8)	HR = 0.9 *P* < .001	24.9 (24.4–25.5)	16.2 (15.7–16.7)	9.9 (9.5–10.3)	HR = 0.92 *P* < .001
		25–34 y	28.7 (28.5–28.9)	19.6 (19.4–19.9)	13.5 (13.3–13.6)	HR = 1.01 *P* = .184	21.9 (21.7–22.1)	12.6 (12.4–12.8)	7.1 (7–7.2)	HR = 1.01 *P* < .001
…		45–54 y	32 (31.7–32.4)	21.6 (21.3–21.9)	14.7 (14.4–14.9)	HR = 0.93 *P* < .001	25.4 (25.1–25.8)	14.5 (14.3–14.8)	7.8 (7.6–8)	HR = 0.93 *P* < .001
		>55 y	32.3 (31.9–32.8)	22 (21.6–22.5)	14.7 (14.3–15)	HR = 0.92 *P* < .001	26.3 (25.9–26.7)	15.5 (15.2–15.9)	8.6 (8.3–8.8)	HR = 0.91 *P* < .001
	Gender	Male	29.9 (29.8–30)	20.3 (20.2–20.4)	13.8 (13.7–14)	Ref Ref	23.3 (23.2–23.5)	13.4 (13.3–13.5)	7.4 (7.3–7.5)	Ref Ref
		Female	31.1 (30–32.3)	22.2 (21.2–23.3)	14.9 (13.9–15.8)	HR = 1.01 *P* = .398	24.3 (23.2–25.3)	14.8 (13.9–15.7)	7.9 (7.2–8.7)	HR = 1.01 *P* = .323
Medicaid	Calendar year	2018	54.8 (53.3–56.4)	45 (43.5–46.6)	37.6 (36.1–39.2)	Ref Ref	28.1 (26.8–29.6)	15.6 (14.5–16.8)	8.1 (7.3–9.1)	Ref Ref
		2019	49.9 (48.7–51.1)	42.1 (41–43.4)	34.3 (33.1–35.5)	HR = 1.14 *P* < .001	21.8 (20.8–22.8)	11.8 (11–12.6)	5.8 (5.2–6.4)	HR = 1.17 *P* < .001
		2020	47.4 (46.2–48.6)	38.4 (37.2–39.6)	30.2 (29.1–31.4)	HR = 1.24 *P* < .001	25.3 (24.3–26.3)	13.1 (12.4–14)	5.5 (4.9–6.1)	HR = 1.08 *P* < .001
		2021	41.4 (40.4–42.4)	32.4 (31.5–33.4)	25.8 (24.8–26.7)	HR = 1.4 *P* < .001	22.7 (21.9–23.6)	11.8 (11.2–12.5)	5.3 (4.9–5.8)	HR = 1.15 *P* < .001
	Age	35–44 y	47.3 (46–48.5)	38.1 (36.9–39.3)	30.5 (29.3–31.7)	Ref Ref	26.3 (25.2–27.4)	14.1 (13.2–15)	6.4 (5.8–7.1)	Ref Ref
		16–24 y	43.7 (42.3–45.2)	35.4 (34–36.8)	27.7 (26.4–29.1)	HR = 1.02 *P* = .4	20.6 (19.5–21.9)	11.5 (10.5–12.4)	5.9 (5.3–6.7)	HR = 1.11 *P* < .001
		25–34 y	48.8 (47.9–49.8)	39.9 (38.9–40.8)	32.1 (31.2–33)	HR = 0.94 *P* < .001	24.4 (23.6–25.3)	13 (12.3–13.7)	6.1 (5.6–6.6)	HR = 1.03 *P* = .089
		45–54 y	48.9 (47.3–50.5)	40.3 (38.7–42)	33.6 (32.1–35.2)	HR = 0.89 *P* < .001	24.6 (23.3–26.1)	13 (12–14.2)	5.6 (4.9–6.4)	HR = 1.03 *P* = .212
		>55 y	42.1 (40–44.3)	33.6 (31.6–35.7)	27.4 (25.5–29.4)	HR = 1.11 *P* < .001	20.6 (18.9–22.4)	9.6 (8.4–11)	3.9 (3.1–4.9)	HR = 1.16 *P* < .001
	Gender	Male	46.8 (46.2–47.5)	38 (37.4–38.6)	30.5 (29.9–31.2)	Ref Ref	23.9 (23.3–24.4)	12.7 (12.2–13.1)	5.8 (5.5–6.2)	Ref Ref
		Female	49 (47.4–50.7)	40.2 (38.6–41.9)	32.7 (31.1–34.4)	HR = 0.95 *P* = .015	24.6 (23.2–26.1)	13.1 (12–14.3)	6.2 (5.4–7.1)	HR = 0.96 *P* = .034
	Race	White	48.7 (47.8–49.6)	40.3 (39.4–41.2)	32.8 (31.9–33.6)	Ref Ref	24.5 (23.7–25.3)	13.6 (13–14.2)	6.6 (6.2–7.1)	Ref Ref
		Black/African American	47 (45.7–48.3)	37.6 (36.3–38.8)	30.4 (29.2–31.6)	HR = 1.1 *P* < .001	24.2 (23.1–25.3)	12.6 (11.8–13.5)	6.1 (5.5–6.8)	HR = 1.01 *P* = .755
		Hispanic/Latinx	32.7 (30.8–34.8)	25.2 (23.4–27.2)	19.1 (17.4–20.9)	HR = 1.53 *P* < .001	18.9 (17.3–20.7)	10.4 (9.2–11.9)	4.5 (3.6–5.5)	HR = 1.19 *P* < .001
		Other races	47.7 (46.2–49.3)	37.9 (36.5–39.4)	30 (28.6–31.4)	HR = 1.07 *P* < .001	24.4 (23.1–25.7)	11.7 (10.7–12.7)	4.8 (4.2–5.5)	HR = 1.04 *P* = .024

Abbreviations: Ag/Ab, antigen/antibody; HR, hazard ratio; PrEP, pre-exposure prophylaxis.

As shown in Supplementary Figure 2, metropolitan areas varied widely with regard to the proportion of PrEP users receiving HIV Ag/Ab testing within 3 and 6 months before PrEP fills. Of the 148 CBSAs evaluated, 117 areas had a rate of PrEP fills without HIV Ag/Ab testing within 3 months of >25%, with 31 areas having rates between 10% and 25%. Likewise, considering HIV Ag/Ab testing within the prior 6 months, 8 areas had a rate of PrEP prescription fills without testing of <10%, 75 areas had a rate between 10% and 25%, and 65 areas had a rate of >25%.

## DISCUSSION

Based on a national cohort including >39 809 PrEP users filling prescriptions from 2018 through 2021, we demonstrated that about 1 in 3 oral PrEP prescriptions were filled in PrEP users who had not received an HIV Ag/Ab test within the prior 3 months, with evidence of health disparities in several key population groups. Moreover, about 1 in 4 oral PrEP prescriptions were filled in persons who had not received any type of HIV testing within the prior 3 months. Study findings can inform efforts to optimize HIV testing compliance with national PrEP guidance.

We identified disparities between key demographic and geographic subgroups. Of note, our finding that PrEP users on their first 3 months of PrEP were significantly more likely to have been tested suggests that some PrEP users may receive guideline-based testing before PrEP initiation but then receive prescription refills without recent testing. We also note that PrEP users with Medicaid insurance appeared to frequently be tested with earlier-generation, Ab-only assays based on the use of billing codes that are for HIV testing but do not specify Ag/Ab testing. While the underlying causes of this Medicaid disparity are uncertain, additional work is needed to assess whether the clinics commonly serving Medicaid-insured PrEP users have more limited access to HIV Ag/Ab assays.

These findings are particularly relevant given the recent update to the CDC Clinical PrEP guidelines in 2021, which recommend HIV RNA NAAT testing in addition to HIV Ag/Ab testing every 3 months for PrEP care monitoring. Given the possible inconsistency of HIV testing during PrEP monitoring before the updated guidelines, we anticipate that testing compliance to the current guidance is low. As HIV RNA NAAT testing was not included in the CDC guidelines during the study period, our finding that PrEP users rarely received HIV RNA NAAT testing was expected; nonetheless, this finding is noteworthy to the extent that the addition of HIV RNA NAAT testing means that the updated guidelines are calling for a major shift in practice.

In addition to providing a comprehensive national view of current testing practices, these findings have many practical applications. Foremost, they highlight the need for clinics and PrEP prescribers to remain attentive to testing needs when refilling prescriptions. Moreover, clinics may wish to incorporate testing reminders for providers and PrEP users into medical record laboratory order sets and clinical workflows as well as include testing compliance in clinic and provider quality improvement measures and feedback audit reporting [21–23]. Prior studies have also highlighted knowledge gaps among PrEP prescribers and the need for education [24–26]. In some cases, the PrEP prescriber might have ordered testing in accordance with CDC guidelines, but the PrEP user did not return for blood draws on schedule. Additional work is needed to determine whether this is a common cause of not testing within CDC guidelines and, if it is, to understand what underlying factors are preventing PrEP users from receiving ordered tests (eg, logistical limitations, such as time away from work, vs a lack of understanding of the importance of HIV testing while on PrEP). If PrEP user factors are a significant barrier to timely testing, implementing testing strategies that improve client convenience, such as sending test orders to phlebotomy sites near the PrEP user's home or work, having evening and weekend hours available for testing at the clinic, and self-testing at home with dried blood spot (DBS) laboratory kits, may prove to be extremely beneficial [26–28]. Moreover, the evidence of disparities that we identified suggests that PrEP users in undertested metro areas and demographic groups may benefit from such targeted initiatives.

The strengths of this study include the large study population comprising a demographically and geographically diverse cohort of PrEP users and the study's important implications for PrEP care. Our findings expand on those previously published by McCormick et al. in 5 significant ways [8]. First, we take a PrEP user–focused view, looking at PrEP user factors that impact testing adherence and rates by prescription fill (as opposed to rates by provider). Second, we differentiate between different types of HIV tests. This is important given that antibody-only testing is suboptimal per guidelines and given the specific addition of NAAT testing to guidelines. Third, we include Medicaid data in addition to commercially insured PrEP users as opposed to commercially insured only. Fourth, we looked at time between recent HIV testing and prescription fills as opposed to testing within specific time windows of initiation and follow-up visits. We think that this approach provides an important perspective; since paralleling guidelines it may not be necessary to retest at a specific visit if the PrEP user otherwise had recent HIV testing. In addition, by looking at 3-, 6-, and 12-month time intervals, we provide a quantitative view of how out of date testing is (eg, filling a prescription without testing in the prior 6 months is worse than filling 1 without testing in the prior 3 months). Finally, we use survival methods to avoid biases related to care before the individual enrolling in the database. To the extent that our findings overlap with those of McCormick et al., our results are highly supportive in that they demonstrate the need for significant improvement efforts with regard to adherence to testing guidelines.

However, this study is also subject to limitations. One potential limitation is the possibility of missing data bias. Most studies based on claims data, including this one, may be subject to biases in the event that an individual receives care that is not billed to insurance and is thus missing from the database. In our study, this could be a particular concern if PrEP users received unbilled HIV testing. Sources of unbilled HIV testing may include some community and student health centers and public health departments, various events attracting large numbers of PrEP users, and certain models of pharmacist-initiated PrEP. We aimed to partly control for this concern by excluding PrEP users without any HIV testing billed to insurance, assuming that these individuals were receiving testing in settings that do not bill. If individuals received both billed and unbilled testing, our analysis would undercount their testing rates. However, unless rates of unbilled testing (in people also receiving billed testing) were much higher than we think is likely, this limitation is unlikely to alter the overall conclusions of this manuscript. In addition, while the data are from a large national cohort, they may not be fully representative of all PrEP users. We did not include Medicare-insured PreP users (ie, PrEP users age >65 years) or uninsured PrEP users; likewise, the Medicaid PrEP users were from selected (and unidentified) states. The study period overlapped with the coronavirus disease 2019 pandemic, which may have affected HIV testing in some PrEP users. However, we do not believe that the pandemic had a major impact on our overall findings given that testing in 2020 was not remarkably different than in the surrounding years (Figure 3).

In conclusion, in a large national sample, we identified that nearly 1 in 3 and 1 in 4 PrEP users received a medication fill without an HIV Ag/Ab test or any HIV test within the prior 3 months, respectively. This information is essential to evaluate testing compliance with national PrEP guidance and to inform stakeholders of what possible immediate interventions are required to roll out PrEP safely and avert undiagnosed infections.

## Supplementary Material

ofae254_Supplementary_Data

## Supplementary Data

Supplementary materials are available at *Open Forum Infectious Diseases* online. Consisting of data provided by the authors to benefit the reader, the posted materials are not copyedited and are the sole responsibility of the authors, so questions or comments should be addressed to the corresponding author.
