# Supplementary material for: High Rates of Missed HIV Testing Among Oral PrEP Users in the United States From 2018–2021: A National Assessment on Compliance With HIV Testing Recommendations of the CDC PrEP Guidelines
Source: Open Forum Infect Dis. 2024 May 23;11(5):ofae254. doi: 10.1093/ofid/ofae254 (PMC11127479; doi:10.1093/ofid/ofae254)
Supplement: ofae254_Supplementary_Data [file ofae254_supplementary_data.docx]

**SUPPLEMENTAL Online Material**

**APPENDIX A: TECHNICAL DEFINITIONS and GEOGRAPHIC MAPPING**

**Age**:  Calendar year of first PrEP fill minus birth year.  (i.e. person’s age prior to birthday during the calendar year the person first received PrEP during the study period)

**Gender:** Person’s gender (only male and female available in database) as of the person’s first year of enrollment in 2016 or later

**Race:**  Standard race as listed in the database (White, Black, Hispanic and other are the only categories listed) as of the person’s first year of enrollment in 2016 or later

**Region:** Person’s region as listed in the database as of the person’s first year of enrollment in 2016 or later.  Only available for commercially insured persons.

**HIV RNA NAAT Quantitative:** Outpatient test billed using CPT 87536

**HIV RNA NAAT Qual:** Outpatient test billed using CPT 87535 or 87538

**HIV RNA NAAT=** HIV RNA NAAT Quantative or HIV RNA NAAT Qual

**HIV Ag/Ab Test:** Outpatient test billed using CPT **87389 or G0475**

**Any HIV Test:** Outpatient test billed with any of the above or 86703 , 87806 , 86701 , 86702 , G0433 , G0435 , G0432 , or 87391 (87391 and 87806 are Ag-only and the others are Ab-only)

**Oral PrEP Medication:** Outpatient prescription with NDC codes "61958070501" "61958200501" "61958200205" "61958070101" "61958200201" "61958200202" "61958070301" "61958070401". We considered each unique person-calendar month in which a PrEP user received oral PrEP as a separate prescription fill (counting at most one prescription per person per calendar month)

**Time between prescription fill and HIV test:** We calculated the number of calendar months between each PrEP prescription fill and the PrEP user’s most recent prior HIV test.  This was taken to be 0 if the PrEP user had an HIV test in the same calendar month as the PrEP prescription fill (regardless of whether the test was before or after the actual prescription dispensation).  If the PrEP user had an HIV test in the calendar month prior to the prescription this would be 1 and so forth.

**Time of first enrollment:** For prescriptions not associated with a prior HIV test we censored cases to the later of January, 2016 or the individual’s time of first continuous enrollment in the database.  The time of first enrollment was the first calendar month prior to the PrEP prescription that the individual was enrolled in coverage at least one day without any subsequent breaks in coverage of at least a full calendar month prior to the PrEP prescription.

**Rural/Urban:** Urban= within MSA, Rural= outside of MSA.   Only available for Commercially insured individuals.  MSA coding is as listed in the database as of the person’s first year of enrollment in 2016 or later.

**EXCLUSION CRITERIA**

**HIV Infection:**  We excluded persons with the following ICD-9/10 codes associated with any outpatient visit in the database.  (regardless of time).  042; 07953; 79571;V08; B20; B9735.

**Other Antiretroviral:** We also excluded persons dispensed HIV medications besides those approved for oral PrEP since these might indicate active HIV infection. We specifically excluded persons who filled a prescription with an NDC code for which the first 9 digits matched one of the following. (A prescription with one of the 11 digit codes for medications approved for oral PrEP would not be an exclusion even if matching one of the 9 digit codes shown in this table).

| keyword | NDC Code (First 9 Digits) |
| --- | --- |
| ABACAVIR | 003784105,009046874,317220557,317220562,423850962,497020206,497020217,497020221,497020222,497020231,497020258,502680049,510790204,643800717,649800405,658620073,658620089,658620335,680840021,681800286,681800288,690970362,690970514,705181274,707101049,707711053,728650167 |
| ALAFENAMIDE | 500906247,596760800,619581901,619582002,619582005,619582101,619582301,619582501,619582505 |
| ATAZANAVIR | 000033622,000033624,000033631,000033638,000033641,000935526,000935527,000935528,167140860,167140861,167140862,317220653,317220654,317220655,423850920,423850921,423850922,658620710,658620711,658620712,658620713,690970443,690970444,690970445,690970446,692381135,692381136,692381137,692381138,707101050,707101051,707101052,707711591,707711592,707711593 |
| BICTEGRAVIR | 500906247,619582501,619582505 |
| CABOTEGRAVIR | 497020240,497020248,497020253,497020264 |
| COBICISTAT | 000033641,500901723,596760575,596760800,619581201,619581401,619581901 |
| DARUNAVIR | 500901723,596760562,596760563,596760564,596760565,596760566,596760575,596760800 |
| DISOPROXIL | 000065007,000935234,000937704,003781930,009047172,155840101,167140534,167140820,317220535,317220560,317220736,333420106,333420138,422910439,422910800,423850915,423850928,423850929,423850953,495020425,495020450,495020475,500905957,502680758,514070112,538081128,596510165,596510166,596510167,602192095,605054202,605054666,606870706,619580401,619580403,619580404,619580405,619580406,619580701,619580703,619580704,619580705,619581101,619581201,621350466,636297581,643800714,643800719,658620354,658620421,658620497,658620818,658620819,658620820,672961440,672961459,672961830,681800287,690760105,690970209,690970210,690970533,690970741,692381527,692381547,692381548,692381549,692382092,692382093,692382094,692382095,705182905,705183677,707101364,707101365,707101366,707101367,707711620,707711621,707711622,707711709,712050776,721890156,721890227,721890312,726060002,762820677,829820056 |
| DOLUTEGRAVIR | 497020226,497020227,497020228,497020231,497020242,497020246,497020255,497020258,538081130 |
| DORAVIRINE | 000063069,000065007,500906237,500906268 |
| EFAVIRENZ | 000560470,000560474,000935234,155840101,317220736,333420138,423850915,423850928,423850929,425430889,495020425,495020475,514070382,606870706,643800889,649800406,649800407,658620049,658620104,658620105,658620106,658620497,690970210,690970301,705182905,705182962,705183258,728650172 |
| ELVITEGRAVIR | 619581201,619581901 |
| EMTRICITABINE | 000935234,000937704,003781930,009047172,155840101,167140534,317220560,317220736,333420106,333420138,422910439,423850915,423850953,500905957,500906247,514070112,596510165,596510166,596510167,596760800,602192095,605054202,606870706,619580601,619580602,619580701,619580703,619580704,619580705,619581101,619581201,619581901,619582002,619582005,619582101,619582501,619582505,636297581,643800719,658620301,658620354,658620497,672961440,672961459,672961830,681800287,690970209,690970210,690970642,690970741,692381527,692381547,692381548,692381549,692382092,692382093,692382094,692382095,705181847,705182905,705182906,707101364,707101365,707101366,707101367,707711620,707711621,707711622,707711709,712050776,721890156,721890227,721890312,721890403,762820677,829820056 |
| ENFUVIRTIDE | 000040381 |
| ETRAVIRINE | 422910473,422910474,596760570,596760571,596760572,602191720,602191721,602191722,692381720,692381721,692381722,693150285,693150286,800050112,800050113 |
| FOSAMPRENAVIR | 003783520,497020207,497020208,633040583 |
| FOSTEMSAVIR | 497020250 |
| IBALIZUMAB | 620640122 |
| LAMIVUDINE | 000065007,001730662,001730663,009046583,317220001,317220506,317220739,317220752,317220753,317220754,333420001,333420002,333420003,423850928,423850929,423850962,430630900,495020425,495020450,495020475,497020202,497020203,497020204,497020205,497020206,497020217,497020231,497020246,497020258,507420623,507420624,548380566,572370274,604290353,604290354,605053250,605053251,605053252,606870362,606870720,643800707,643800710,643800711,658620025,658620026,658620036,658620055,658620335,669930478,680714450,681800284,681800286,681800288,681800602,681800603,690970166,690970167,690970362,707101049,707711053,713350141,713350639,713351916,726060002,728650144,762820115 |
| LENACAPAVIR | 619583001,619583002 |
| LOPINAVIR | 000740522,000741575,000742605,000743956,000746799,005271947,317220556,317220603,423850933,423850934,680712949 |
| MARAVIROC | 317220579,317220580,497020223,497020224,497020233,497020235,497020237,497020260,500906238 |
| NELFINAVIR | 630100010,630100027 |
| NEVIRAPINE | 003784050,003784890,005970047,317220505,333420004,333420238,425710131,643800709,651620209,658620027,658620057,658620932,658620933 |
| RALTEGRAVIR | 000060227,000060473,000060477,000063080,000063603,500901085,680712113,712050777,829820055 |
| RILPIVIRINE | 497020240,497020242,497020253,596760278,619581101,619582101 |
| RITONAVIR | 000540407,000690345,000691085,000691101,000740522,000741575,000742340,000742605,000743093,000743333,000743399,000743956,000746799,005271947,317220556,317220597,317220603,423850933,423850934,538081119,606870420,651620061,658620687,680712949,690970655 |
| SAQUINAVIR | 000040244 |
| SOFOSBUVIR | 619581501,619581503,619581504,619581505,619581801,619581803,619581804,619581805,619582201,619582203,619582204,619582205,619582401,726262601,726262701 |
| STAVUDINE | 531040111 |
| TENOFOVIR | 000065007,000935234,000937704,003781930,009046821,009047172,155840101,167140534,167140820,317220535,317220560,317220736,333420096,333420106,333420138,422910439,422910800,423850915,423850928,423850929,423850953,495020425,495020450,495020475,500905957,500906247,502680758,514070112,538081128,596510165,596510166,596510167,596760800,602192095,605054202,605054666,606870706,619580401,619580403,619580404,619580405,619580406,619580701,619580703,619580704,619580705,619581101,619581201,619581901,619582002,619582005,619582101,619582301,619582501,619582505,621350466,636297581,643800714,643800719,658620354,658620421,658620497,658620818,658620819,658620820,672961440,672961459,672961830,681800287,690760105,690970209,690970210,690970533,690970741,692381527,692381547,692381548,692381549,692382092,692382093,692382094,692382095,705182905,705183677,707101364,707101365,707101366,707101367,707711620,707711621,707711622,707711709,712050776,721890156,721890227,721890312,726060002,762820677,829820056 |
| TIPRANAVIR | 005970003 |
| VELPATASVIR | 619582201,619582203,619582204,619582205,619582401,726262701 |
| ZIDOVUDINE | 317220506,317220739,333420003,430630900,497020202,497020211,497020212,497020213,497020217,643800707,658620024,658620036,658620048,658620107,680714450,681800284,681800286,705181656,713350141,713350639,713351916,728650144,762820115 |

**HBV Infection:** We excluded individuals with the following ICD-9/10 codes associated with any outpatient visit in the database.  (regardless of time).  07020; 07021; 07022; 07023; 07030; 07031

**Age Exclusion:** We excluded individuals who were younger than 16 (based on the definition of age above)

**Geographic Mapping Approach:**

We developed the maps using ggplots2 and relevant polygon coordinates (latitudes and longitudes). PrEP users were linked to their corresponding CBSA based on the MSA code shown in the database. Since CBSAs are comprised of one or more MSAs, we used a crosswalk downloaded from census.gov to determine each individual’s CBSA. Geographic coordinates for CBSA polygons were derived from the coordinates of each CBSAs underlying counties with county coordinates obtained from the R maps package. A crosswalk between CBSAs and county (FIPS) was downloaded from public.opendatasoft.com. Polygon coordinates for the US and US states obtained from the R “maps” package. Coordinates for cities were downloaded from simplemaps.com.

**Supplemental Figures**

Figure S1: Analog to Figure 2 with One PrEP Fill Sampled per PrEP User

**
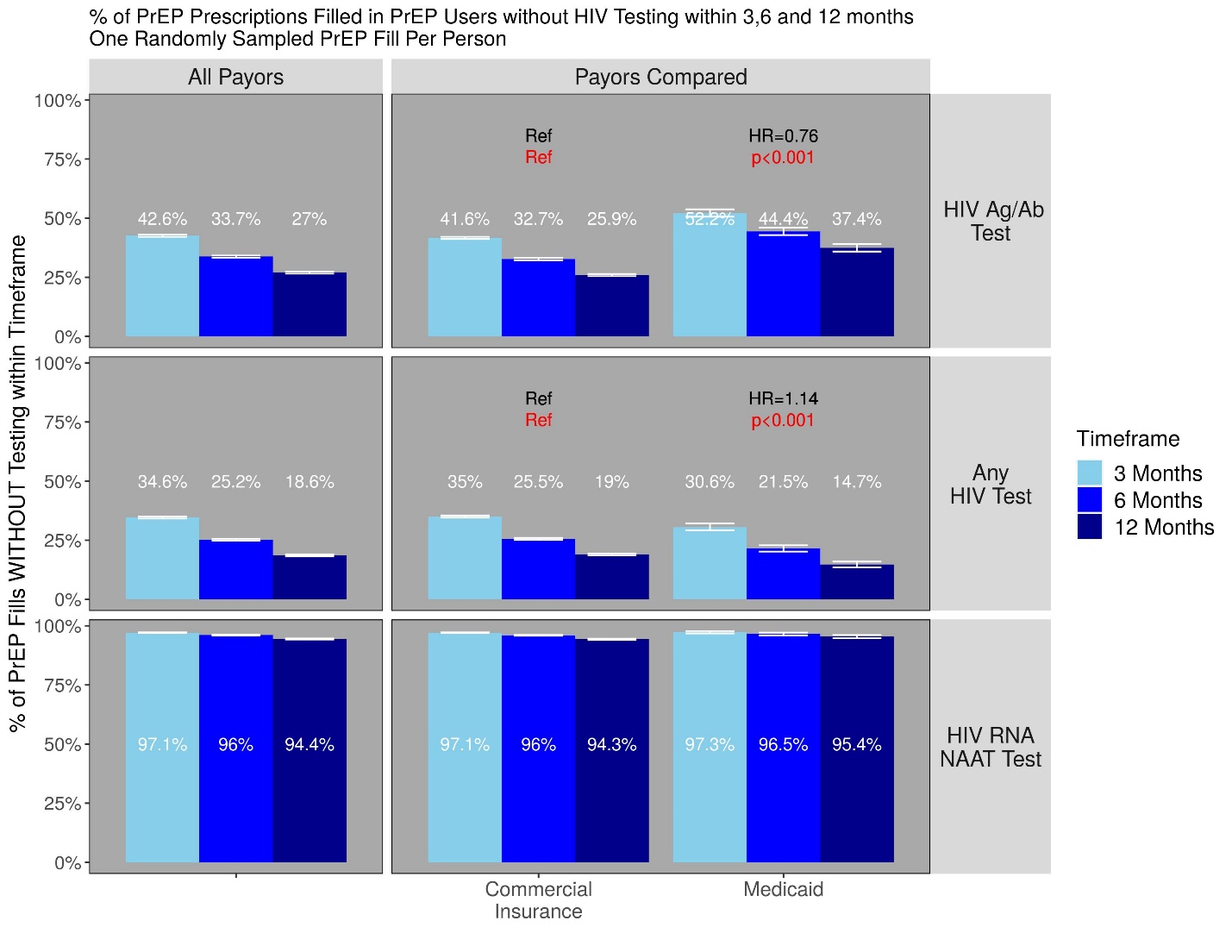
**

Supplemental Figure S2: Variation in Patterns of HIV Testing for PrEP Users by Metropolitan Area

**
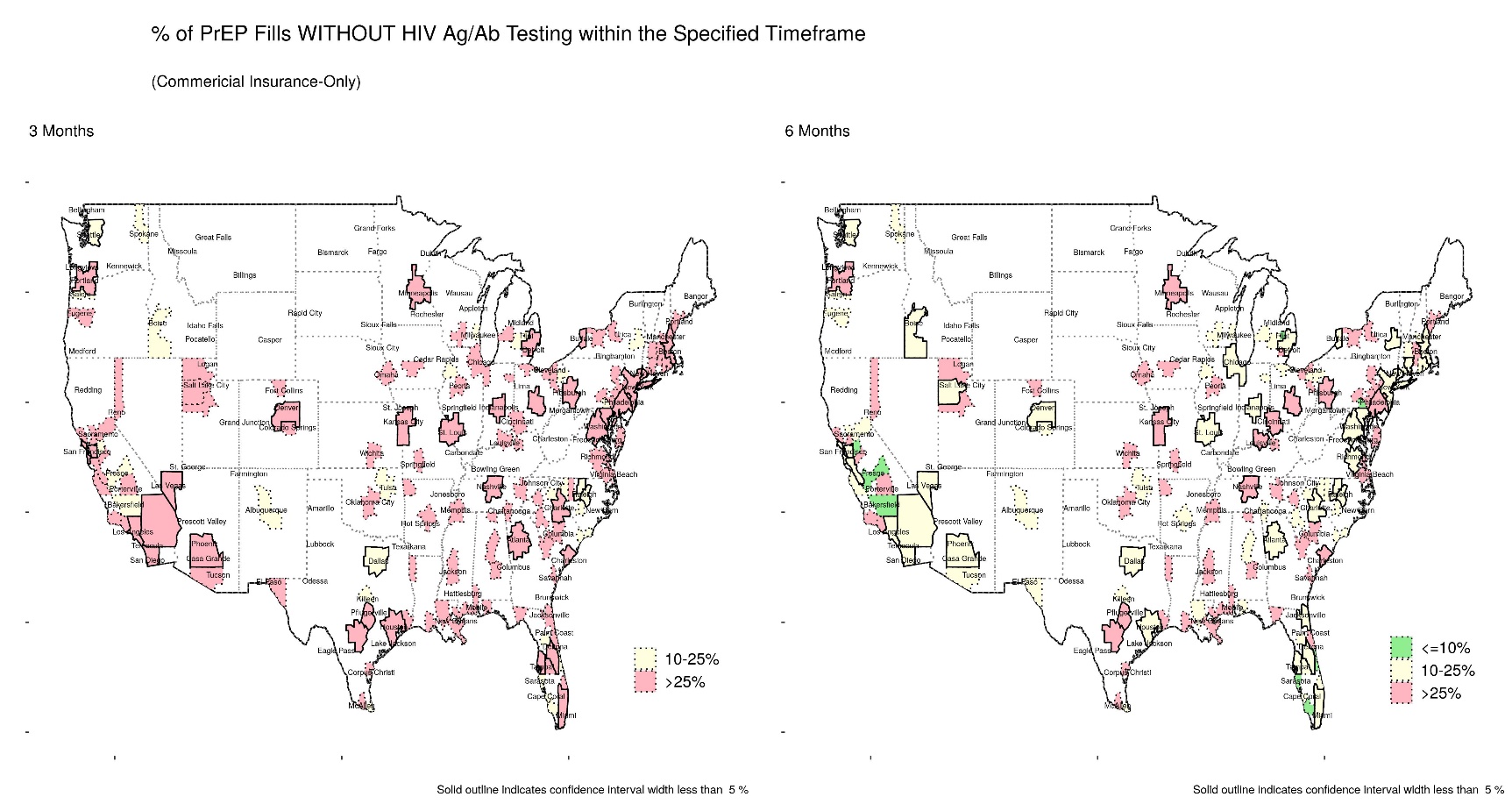
**

Shown are maps of the continental United States with the most populous CBSAs colored according to the proportion of PrEP prescription fills with HIV testing as specified. White areas represent areas outside of CBSAs or CBSAs not specifically included in the analysis. CBSAs with rates significantly different from the national mean are outlined with a solid line; dashed outlines indicate lack of statistical significance.
